# Determination of toxic (Pb, Cd) and essential (Zn, Mn) metals in canned tuna fish produced in Iran

**DOI:** 10.1186/s40201-015-0215-x

**Published:** 2015-08-11

**Authors:** Seyed Vali Hosseini, Soheil Sobhanardakani, Hamed Kolangi Miandare, Mohammad Harsij, Joe Mac Regenstein

**Affiliations:** Department of Fisheries, College of Agriculture & Natural Resources, University of Tehran, Karaj, Iran; Department of the Environment, College of Basic Sciences, Hamedan Branch, Islamic Azad University, Hamedan, Iran; Department of Fisheries, Gorgan University of Agricultural Sciences and Natural Resources, Gorgan, Iran; Department of Natural Resources, Gonbad Kavous University, Gonbad Kavous, Iran; Department of Food Science, Cornell University, Ithaca, New York USA

**Keywords:** Canned fish, Food safety, Lead, Cadmium, Zinc, Manganese

## Abstract

**Background:**

Metal pollution of waterways directly affects human health and can impact the food chain. Seafood living in polluted water can accumulate trace metals. The purpose of this study was to analyze the toxic metals Pb and Cd and the dietary essential metals Zn and Mn in 120 cans of tuna species from four different brands processed in Iran and purchased in 2012.

**Results:**

The mean level of metals for each brands of canned fish obtained in mg/kg were as follows: yellowfin tuna (Pb: 0.19 ± 0.015, Zn: 5.77 ± 4.17, Mn: 0.08 ± 0.07, Cd: 0.15 ± 0.12), Kilka (Pb: 0.95 ± 0.88, Zn: 30.47 ± 29.82, Mn: 1.01 ± 0.73, Cd: 0.07 ± 0.05), Kawakawa (Pb: 0.28 ± 0.23, Zn: 6.77 ± 5.21, Mn: 0.17 ± 0.12, Cd: 0.12 ± 0.09), longtail tuna (Pb: 1.59 ± 1.56, Zn: 7.44 ± 6.11, Mn: 0.04 ± 0.03, Cd: 0.06 ± 0.04). Pb, Zn and Cd levels were generally higher than the FAO/WHO permissible limits (Pb: 0.50 mg/kg, Zn: 50.0 mg/kg and Cd: 0.50 mg/kg) and the European Union acceptable dietary limits.

**Conclusions:**

Based on the United States Environmental Protection Agency health criteria, there is no health risk associated with Mn concentration in the samples analyzed. The limits of detection of the method for Pb, Zn, Mn and Cd in mg/kg were 0.01, 0.5, 0.01 and 0.01, respectively. The result of the one-way analysis of variance suggested significant variations (p < 0.05) in the concentration of the metals in the different types of canned fish with the following being outside of compliance levels.

## Introduction

During the last few decades there has been a growing interest in determining the level of toxic metals in marine and fresh water environments with additional emphasis on the measurement of contamination levels in the food supply, particularly fish [1–12] including canned fish. For example, Tuzen and Soylak determined the Cu, Zn, Mn, Fe, Se, Al, Cr, Ni, Pb and Cd concentrations in canned fish marketed in Turkey [1]. Mol analyzed the levels of Fe, Zn, Cu, Cd, Sn, Hg and Pb in canned bonito, sardines, and mackerel [5], canned tuna fish [6] and canned anchovies and canned rainbow trouts [7] produced in Turkey. Hosseini et al. determined Hg, Se and Sn concentrations in canned fish marketed in Iran [10] while Mahalakshmi et al. determined Al, Cd, Pb and Hg in canned tuna fish available in Canada and India [11]. Trace metals are important for both their necessity and toxicity. Some elements like Mn and Zn are essential functional and structural elements in biological systems [13–18] often catalyzing reactions by binding to substrates, there by favoring various reactions such as the mediation of oxidation–reduction reactions, or redox reactions, through reversible changes in the oxidation state of the metal ions [15, 16, 19–21]. For Mn and Zn, often called micronutrients, there are fixed allowed levels that provide for an adequate dietary intake according to the World Health Organization (WHO). In adults from 5.0 to 22.0 mg are recommended for Zn and from 2.0 to 20.0 mg are recommended for Mn [22, 23]. At high concentrations, Zn causes nephritis, anuria and extensive lesions in the kidneys [24, 25].

Pb and Cd are very toxic to humans. They are only tolerated at extremely low concentrations and excesses are associated with many adverse health effects [12, 26]. They may injure the kidney and cause symptoms of chronic toxicity, including impaired organ function, poor reproductive capacity, hypertension, tumors and hepatic dysfunction [24]. Moreover, Pb can also affect brain function by interfering with neurotransmitter release and synapse formation. Exposure to Pb has been associated with reduced IQ, learning disabilities, slow growth, hyperactivity, antisocial behaviors and impaired hearing [27]. Generally Pb-poisoning is ranked as the most common environmental health hazard [28].

Use of Cd in agriculture and industry has been identified as a major source of its wide dispersion in the environment and food. The major route of exposure to Cd for non-smokers is via food; the contribution from other pathways to total uptake is small [28]. Certain marine vertebrates contain markedly elevated Cd concentrations [29].

Trace and toxic metals may contaminate fish, mainly during the growth phase but also, sometimes, due to contamination during transportation and storage. Therefore, most countries monitor the levels of toxic metals in seafood [5, 30, 31]. Levels of toxic metals in fish depend on many factors like the duration of exposure of fish to contaminants in the water, the feeding habit of the fish, the concentrations of contaminants in the water column, and sometimes to the water chemistry, contamination of fish during handling and processing, age, sex, weight, season, fish species, catching area, transportation, and storage, etc. [21, 32, 33]. According to Taha’n et al., the pH of the canned product, the quality of the lacquer coatings of canned products, oxygen concentration in the headspace, quality of the coating and storage place may also affect metal levels in canned fishes [34].

Many species of commercially caught marine fish, especially in Iran, are canned, thus making them more available for human consumption to those living far from the sea [35].

Tuna fish are long living organisms prone to accumulate pollutants. Canned tuna fish is eaten regularly in many countries including Iran (globally over the 10 × 10^10^ tonne per year) [12, 36–39]. In this study the levels of the toxic and essential metals (Pb, Zn, Mn and Cd) in four different commercial types of canned tuna fish (longtail tuna, Kawakawa, Kilka and yellowfin tuna) commonly consumed in Iran were determined using GFAAS . This study will help to generate the data needed for surveillance programs aimed at ensuring the safety of the food supply and minimizing human exposure to toxic metals.

## Materials and methods

### Sample collection

During the year 2012, 120 samples (185 g each) of four different commercial types of canned fish commonly consumed in Iran (30 samples for each type: “Famila Co.” (Tehran): yellowfin tuna (YT) (*Thunnus albacares*); “Shilaneh Co.” (Qazvin): common Kilka (CK) (*Clupeonella cultriventris* caspia); “Pars Tuna Co.” (Bushehr): Kawakawa (Ka) (*Euthynnus affinis*), and “Hiltune Co.” (Tehran): longtail tuna (LT) (*Thunnus tonggol*)) were purchased at markets within Tehran.

### Chemical analyses

All glassware was cleaned by soaking overnight in 10 % nitric acid, followed by rinsing with distilled water. The acids used for wet digestion were of analytical reagent (Merck, Darmstadt, Germany) grade, while the distilled water was further deionized. The blank values were below the detection limits of the instrument. Working standards were made from the stock by dilution of the measured aliquots with 1.0 M nitric acid. Each sample was analyzed in triplicate and the results, which mostly agreed within ±1.0 %, were averaged. A reagent blank determination was carried out with every batch of 10 samples.

After opening each can oil/broth was drained off and the meat was homogenized thoroughly in a food blender (Hongdun HWT, Zhangqiu, China). Samples were then digested without delay. About 5 g of each sample was digested in a quartz Erlenmeyer flask with 15 ml of suprapure nitric:perchloric:sulphuric acid (25 + 25 + 1 v:v:v) mixture, using a hot plate at 150 °C. Further aliquots of nitric acid were added until a complete colorless solution was obtained. After evaporation using the Perkin Elmer Multiwave 3000, the residue was dissolved in 10 ml of water with 1 ml of concentration of suprapure HCl at 100 °C. Finally, the volume was made up to 25 ml with deionized-distilled water. Determination of Pb and Cd were done on a stabilized temperature graphite furnace atomic absorption spectrometer (GFAAS) (4110 ZL, Perkin Elmer). Zn and Mn were done by direct aspiration of the sample solution into the air-acetylene flame [38]. All analyses were performed with three replications. The recovery of various metals from canned fish samples is presented in Table 1.

**Table 1:** Recovery of various metals from canned fish samples

Sample	Pb			Zn			Mn			Cd		
	Added (mg/kg)	Found (mg/kg)	Recovery (%)	Added (mg/kg)	Found (mg/kg)	Recovery (%)	Added (mg/kg)	Found (mg/kg)	Recovery (%)	Added (mg/kg)	Found (mg/kg)	Recovery (%)
YT	30	30	100	30	29.5	98.3	30	29.9	99.6	30	28.5	95.0
CK	30	29.5	98.3	30	29.8	99.3	30	29.5	98.3	30	29.3	97.6
Ka	30	29.3	97.6	30	28.0	93.3	30	30	100	30	29.3	97.6
LT	30	28.8	96.0	30	29.9	99.6	30	29.5	98.3	30	28.9	96.3

### Statistical analysis

One-way analyses of variance (ANOVA) and Tukey’s test were used to determine whether Pb, Zn, Mn and Cd concentrations varied significantly between specimens, with probability values less than 0.05 (p < 0.05) considered statistically significant. The statistical calculations were done using SPSS 15.0 version (SPSS Inc., Chicago, IL, USA) statistical package.

## Results

The concentrations of Pb, Zn, Mn and Cd in canned fish are presented in Table 2 along with relevant statistical parameters. Thirty samples for each canned fish were analyzed. Cadmium mean concentration in all samples analyzed exceed the EU limit of 0.05 mg kg^−1^, whereas Pb levels in 90 of 120 samples (75.0 %) exceeded the EU acceptable limit of 0.2 mg/kg [40].

**Table 2:** Metals content (mg/kg) for various species of canned fish*

Metal	Yellowfin tuna		Kilka		Kawakawa		Longtail tuna	
	Range	Mean ± SD	Range	Mean ± SD	Range	Mean ± SD	Range	Mean ± SD
Pb	0.02-0.93	0.19 ± 0.015^a^	0.02-3.77	0.95 ± 0.88^c^	0.02-1.17	0.28 ± 0.23^b^	0.07-5.50	1.59 ± 1.56^d^
Zn	4.05-8.22	5.77 ± 4.17^a^	24.72-36.22	30.47 ± 29.82^d^	4.92-10.02	6.77 ± 5.21^b^	4.60-13.37	7.44 ± 6.11^c^
Mn	0.02-0.40	0.08 ± 0.07^b^	0.35-1.40	1.01 ± 0.73^d^	0.02-0.37	0.17 ± 0.12^c^	0.02-0.30	0.04 ± 0.03^a^
Cd	0.02-0.27	0.15 ± 0.12^d^	0.02-0.20	0.07 ± 0.05^b^	0.000-0.27	0.12 ± 0.09^c^	0.02-0.37	0.06 ± 0.04^a^

*Number of each canned fish sample = 30

Vertically, letters a, b, c and d show statistically significant differences of metals content between canned fish specimens according to one way ANOVA analysis (p < 0.05)

The comparative levels based on the average of Pb, Zn, Mn and Cd in the various types of canned fish shows that the average concentrations of Pb in LT are higher than the other species by 1.7, 5.7 and 8.3 times as compared to CK, Ka and YT, respectively. The average Zn content in CK was much higher than other the canned fish, as compared to LT, Ka and YT, respectively. Similar behavior is shown by Mn. The average Pb content in LT was much higher than other the canned fish, as compared to YT, Ka and CK, respectively. The average Cd content in LT was about 3 times lower than YT.

## Discussion

Eboh et al. reported that Pb in the muscle, gills and liver tissue of five common commercially available fish species in Nigeria (catfish, tilapia, ilisha, bonga and mudskipper) were found in the range of 0.001-0.002 mg/kg but did not find any toxic metal residues in salmon and mackerel species [41]. On the other hand, the mean concentrations of Cd (0.37-0.79 mg/kg) and Pb (4.27-6.12 mg/kg) reported by Canli and Atli in muscle tissues of six different fish species (*Sparus auratus*, *Atherina hepsetus*, *Mugil cephalus*, *Trigla cuculus*, *Sardina pilchardus* and *Scomberesox saurus*) are higher than the current results [42]. Turkmen et al. determined the metal levels in the muscles and livers of 12 fish species from the Aegean and Mediterranean Seas and reported that the levels of Cd, Mn, Pb and Zn in muscles of fish were <0.01-0.39, 0.18-2.78, 0.21-1.28 and 3.51-53.5 mg/kg, respectively [43]. Boadi et al. determined Pb, Zn, Fe, Cd, Mn and Hg in 46 canned fish samples of nine different brands marketed in Ghana. The trace metals were found in the range of 0.058- 0.168 mg kg^−1^ for Pb, 0.010-0.370 mg/kg for Zn, 0.001-0.057 mg/kg for Mn and Cd concentration, were below their detection limit for all the samples. Zn levels were generally below the FAO recommended limit of 40 mg kg^−1^. The concentration of Pb in the canned fish was also below the MAFF (1995) guidelines of 2.0 mg kg^−1^ [33, 44]. Also, based on the U.S. EPA health criteria for carcinogens, there are no health risks associated with Pb concentrations in the canned fishes they analyzed [33]. The Cd concentrations in tuna fish samples from the Persian Gulf area of Iran were reported as 0.0046-0.0720 mg/kg with mean values of 0.0223 mg/kg while the Pb concentration ranged as 0.0162-0.0726 mg/kg with mean values of 0.0366 mg/kg [12]. These were lower than the current results. Comparison of the results of this study with other studies is shown in Table 3.

**Table 3:** Comparison of present mean values in specimens with other studies result

Specimen/Fish Species	Area	Tissue	Pb (mg/kg)	Zn (mg/kg)	Mn (mg/kg)	Cd (mg/kg)
Yellowfin tuna, Kilka, Kawakawa, longtail tuna	Present Study	Muscle	0.02-5.50	4.05-36.22	0.02-1.40	0.00-0.27
*Anguilla Anguilla*, *Mugil cephalus*, *Oreochromis niloticus*	Turkey^a^	Muscle	0.21-1.45	34.76-134.76	2.34-18.97	0.04-0.23
		Liver	0.56-2.10	1.28-587.23	4.12-23.81	0.11-4.19
		Gills	0.23-2.36	1.28-169.87	4.39-753.74	0.01-0.52
*Otolithes ruber, Pampus argenteus*, *Parastromateus niger, Scomberomorus commerson, Onchorynchus mykiss*	Iran^b^	Muscle	0.007-0.075	0.005-0.40		0.001-0.45
		Liver	0.005-0.10	0.012-0.45		0.002-0.58
		Gills	0.031-0.11	0.03-0.062		0.004-0.09
Canned salmon, sardine and tuna fish	KSA ^c^	Muscle	0.03-1.97	3.80-23.90		0.01-0.69
Canned fish	USA^d^		0.0-0.03	0.14-97.8	0.01-2.55	0.0-0.05
Canned sardines	Nigeria^e^			0.09-4.63	0.64-1.37	0.11-0.26
Canned tuna	Turkey^f, g, h^		0.09-0.45	8.20-12.4		0.01-0.02
Canned tuna			0.09-0.45	8.20-12.40		0.01-0.02
Canned anchovies, canned rainbow trout			0.178	17.06		0.145
Canned tuna	Canada^i^		0.011-0.089			0.020-0.025

^a^Yilmaz [49]

^b^Sobhanardakani et al. [50, 51]

^c^Ashraf et al. [37]

^d^Ikem and Egeibo [38]

^e^Iwegbue et al. [25]

^f^Mol [5]

^g^Mol [6]

^h^Mol [7]

^i^Mahalakshmi et al. [11]

Several organizations, such as the FAO and WHO, provide guidelines on the intake of trace elements by humans. The provisional tolerable weekly intake (PTWI) recommended by the Joint FAO/WHO Expert Committee (1972) for Cd and Pb are 7 μg Cd/kg body weight per week and 25 μg Pb/kg body weight per week respectively [45, 46]. Therefore, the provisional tolerable weekly intake of Cd and Pb for a 60 kg adult (halfway between the 70 kg male and 50 kg female normally taken as the standard) was estimated to be 420 and 1500 μg/person/week, respectively. The maximum Cd level found in this study was 0.37 mg/kg and therefore a 60 kg adult could safely consume 1135 g portions of fish per week with that level. The maximum Pb concentration observed was 5.50 mg/kg and thus consumption of more than 272 g of fish per week exceeds the tolerable weekly intake of Pb. Toxic metal concentrations in this study were considerably higher than those found previously found in fish by Ikem and Egeibo; Iwegbue et al.; Mol; Boadi et al. and Mahalakshmi et al. [5, 11, 25, 33, 38].

## Conclusion

The results of this study suggested that significant differences existed in the element concentrations across four different species of canned fish. Also, analytical data obtained from this study shows that the metal concentrations for the varieties of canned fishes especially Cd and Pb were generally higher than the FAO/WHO, FDA and U.S. EPA recommended limits for fish [47, 48]. Therefore both low-risk groups (adolescents and adults) and high-risk groups (pregnant mothers and children) must, based on the results obtained, reduce their consumption of canned fish as frequent consumption may result in bioaccumulation of the metals and increased health risks. Globally, further reduction in the levels of environmental contaminants emanating from power plants and other industrial emissions and effluent discharges are needed to reduce contaminant inputs into the aquatic environment. More research and assessments of seafood quality is needed in many countries to provide more data and help safeguard the health of humans. Therefore, it was concluded that toxic metals in canned fish must be monitored comprehensively and periodically with respect to the consumer health.
